# Complete Genome Sequence of Steroid-Transforming Nocardioides simplex VKM Ac-2033D

**DOI:** 10.1128/genomeA.01406-14

**Published:** 2015-01-08

**Authors:** Victoriya Y. Shtratnikova, Mikhail I. Schelkunov, Yury A. Pekov, Victoria V. Fokina, Mariya D. Logacheva, Sergey L. Sokolov, Eugeny Y. Bragin, Vasily V. Ashapkin, Marina V. Donova

**Affiliations:** a Department of Bioengineering and Bioinformatics, Lomonosov Moscow State University, Moscow, Russia; b G.K. Skryabin Institute of Biochemistry & Physiology of Microorganisms, Russian Academy of Sciences, Pushchino, Russia; c A.N. Belozersky Department of Bioengineering and Bioinformatics, M.V. Lomonosov Moscow State University, Moscow, Russia

## Abstract

Nocardioides simplex VKM Ac-2033D is an effective microbial catalyst for 3-ketosteroid 1(2)-dehydrogenation, and it is capable of effective reduction of carbonyl groups at C-17 and C-20, hydrolysis of acetylated steroids, and utilization of natural sterols. Here, the complete genome sequence is reported. An array of genes related to steroid metabolic pathways have been identified.

## GENOME ANNOUNCEMENT

Nocardioides simplex VKM Ac-2033D (synonyms, Arthrobacter simplex [basonym] and Pimelobacter simplex [senior homotypic synonym] [1]) effectively introduces a 1(2)-double bond in various 1(2)-saturated 3-ketosteroids, thus enabling the production of valuable pharmaceuticals and immediate precursors for the steroid industry (2–4). The strain is also capable of effective hydrolysis of acetylated steroids (3), utilization of natural sterols, and the reduction of carbonyl groups at C-17 and C-20 of androstanes and pregnanes, respectively. This bacterium of soil origin was first classified as Arthrobacter globiformis 193 and then reclassified as N. simplex VKM Ac-2033D based on a complex analysis using a polyphase taxonomic approach (2).

The short-read library containing DNA fragments of 226 ± 33-bp insert length was prepared with a TruSeq DNA sample preparation kit (Illumina) after digestion of the genomic DNA with NEBNext double-stranded DNA (dsDNA) fragmentase. The library was read on a HiSeq 2000 (with paired-end 100-nucleotide reads). The mate-pair libraries with 3,222 ± 251-bp-long to 9,992 ± 2,172-bp-long fragments were created with the Nextera mate-pair sample preparation kit (Illumina) and were sequenced on a MiSeq. NextClip 0.8 (5) was used to remove possible paired-end contaminations. Both the paired-end and mate-pair reads were adapter and quality trimmed by Trimmomatic 0.32 (6). The mean coverage of the genome by three libraries was 1,989×. *De novo* genome assembly was performed with Velvet 1.2 (7) and SPAdes 2.5 (8) using paired-end reads and with SPAdes 3.1.0, CLC Genomics Workbench 6.0, and MaSuRCA 2.3.2 (9) using both paired-end and mate-pair reads. The produced contigs were manually combined into a single circular contig in BioEdit (10). The quality of the resulting contig was assessed by REAPR 1.0.17 (11). The contig was also checked by mapping reads in CLC Genomics Workbench and by a visual inspection of putatively ambiguous places.

The length of the genome is 5,637,355 nucleotides (nt), and the G+C content is 72.66%. Annotation of the genome was carried out with the service RAST (http://rast.nmpdr.org/) and with GenBank tools. The RAST annotation revealed 5,421 protein-coding sequences, and the GenBank annotation revealed 4,633 coding sequences (CDS) and 816 pseudogenes; both annotations show 46 tRNAs (44 of which were unique), one pseudo-tRNA and 6 rRNAs. A preliminary analysis of the sequences showed several clusters of genes involved in cholesterol metabolism (side chain degradation, steroid core degradation, and transport).

The reported complete genome sequence will contribute to the elucidation of the range of the steroid substrates that may be metabolized by this organism and the revelation of the scope of its potential application in pharmaceutical steroid production.

### Nucleotide sequence accession number.

The complete genome sequence has been deposited in GenBank under the accession no. CP009896.
